# Do Patient-Reported Upper-Body Symptoms Predict Breast Cancer-Related Lymphoedema: Results from a Population-Based, Longitudinal Breast Cancer Cohort Study

**DOI:** 10.3390/cancers14235998

**Published:** 2022-12-05

**Authors:** Sandra C. Hayes, Matthew Dunn, Melanie L. Plinsinga, Hildegard Reul-Hirche, Yumeng Ren, E-Liisa Laakso, Melissa A. Troester

**Affiliations:** 1 Menzies Health Institute Queensland, Griffith University, Brisbane 4111, Australia; 2 School of Health Sciences and Social Work, Griffith University, Brisbane 4111, Australia; 3 Department of Epidemiology, Gillings School of Global Public Health, University of North Carolina, Chapel Hill, NC 27599, USA; 4 Royal Brisbane and Women’s Hospital, Physiotherapy Department, Brisbane 4029, Australia; 5 Mater Research, South Brisbane 4101, Australia

**Keywords:** breast cancer, symptoms, upper-body function, lymphoedema, cohort study

## Abstract

**Simple Summary:**

Using data from 2442 women with invasive breast cancer, we explored the relationship between upper-body symptoms and upper-body function, breast cancer-related lymphoedema (BCRL), physical activity levels, and quality of life, and assessed whether the presence of upper-body symptoms predicts BCRL. We measured women at three time-points: baseline (between 2- and 9-months post-diagnosis), and at 2- and 7-years post-diagnosis. Upper-body symptoms are common post-breast cancer, and persist into longer-term survivorship. The presence of symptoms is associated with poorer upper-body function, and lower physical activity levels and quality of life. The presence of one or more symptoms of moderate severity or higher at baseline is associated with increased odds of developing BCRL by 2- and 7-years post-diagnosis, with the higher number of symptoms associated with higher odds.

**Abstract:**

The objectives of this work were to (i) describe upper-body symptoms post-breast cancer; (ii) explore the relationship between symptoms and upper-body function, breast cancer-related lymphoedema (BCRL), physical activity levels, and quality of life; and (iii) determine whether the presence of upper-body symptoms predicts BCRL. Nine symptoms, upper-body function, lymphoedema, physical activity, and quality of life were assessed in women with invasive breast cancer at baseline (2- to 9-months post-diagnosis; n = 2442), and at 2- and 7-years post-diagnosis. Mann–Whitney tests, unpaired t-tests, and chi-squared analyses were used to assess cross-sectional relationships, while regression analyses were used to assess the predictive relationships between symptoms at baseline, and BCRL at 2- and 7-years post-diagnosis. Symptoms are common post-breast cancer and persist at 2- and 7-years post-diagnosis. Approximately two in three women, and one in three women, reported >2 symptoms of at least mild severity, and of at least moderate severity, respectively. The presence of symptoms is associated with poorer upper-body function, and lower physical activity levels and quality of life. One or more symptoms of at least moderate severity increases the odds of developing BCRL by 2- and 7-years post-diagnosis (*p* < 0.05). Consequently, improved monitoring and management of symptoms following breast cancer have the potential to improve health outcomes.

## 1. Introduction

Breast cancer is the most commonly diagnosed cancer among women worldwide [1]. The majority of women, particularly those living in high-income countries diagnosed with early-stage disease [2], can expect a good prognosis. With an overall 5-year survival rate of >90% [3], the need to better understand the detrimental, long-term side-effects of breast cancer treatment is clear. Of these, breast cancer-related lymphoedema (BCRL) is one of the most common and feared side-effects [4]. 

Breast cancer-related lymphoedema first presents as an increase in extracellular fluid in the upper limb, breast, or trunk on the treated side [5]. Progression of BCRL involves visible size changes to the affected area and the later deposition of fatty and fibrotic tissue. Once it develops, BCRL is intractable [5], costly, and time-consuming to manage [6], adversely affects quality of life, and is associated with impaired upper body function, lower levels of physical activity, and poorer survival [7,8,9,10]. Despite breast cancer treatment becoming less invasive and more targeted over the past two decades, BCRL remains prevalent with approximately one in five women developing lymphoedema within the first 24 months post-breast cancer diagnosis [11,12]. 

Those with BCRL report a higher prevalence of upper-body symptoms (e.g., pain, tightness, heaviness) compared with women without BCRL [9]. Anecdotally, clinicians indicate that women with BCRL frequently report changes in, or new, upper-body symptoms prior to their definitive diagnosis of lymphoedema. These observations are also reflected in lymphoedema staging definitions reported in the consensus statement of the International Society of Lymphology [5]. Specifically, Stage 0 lymphoedema is characterised as a subclinical or latent condition whereby alterations to lymph transport influence subjective upper-body symptoms such as tightness and heaviness in the absence of measurable swelling [5]. This latent stage may be transitory, or may exist months or years before measurable swelling occurs and progresses to lymphoedema stages I–III [5]. Consequently, it seems plausible that the presence of upper-body symptoms could predict BCRL, and therefore be used to identify those who may benefit most from potential lymphoedema prevention strategies such as compression or exercise therapy [13,14]. The purpose of this work was to (i) describe upper-body symptoms within the first 2 years post-breast cancer diagnosis; (ii) explore the relationship between upper-body symptoms and upper-body function, BCRL, physical activity levels and quality of life; and (iii) determine whether the presence of upper-body symptoms within the first 9 months post-breast cancer diagnosis predicts BCRL at 2- and 7-years post-diagnosis of breast cancer. 

## 2. Materials and Methods

The Carolina Breast Cancer Study Phase 3 (CBCS3) is a longitudinal study that was initiated to evaluate patterns of survivorship following diagnosis. The study involves prospective follow-up of a population-based sample of women with invasive breast cancer based in 44 counties in eastern and central North Carolina, USA (*n* = 2998). Eligible women were English-speaking, 20–74 years old, and newly diagnosed with invasive breast cancer. Younger women (<50 years old) and Black women were oversampled to represent approximately 50% of the study population [15]. Women were identified through the North Carolina Central Cancer Registry between 2008 and 2013 and invited to participate within two months following their diagnosis [16]. The protocol for the study was approved by the University of North Carolina School of Medicine Institutional Review Board. Informed consent was obtained from all participating women. This study is reported following the Strengthening the Reporting of Observational Studies in Epidemiology (STROBE) guidelines for cohort studies [17]. 

Baseline data collection occurred via an in-person interview by trained nurses by 9 months post-diagnosis (median: 5 months post-diagnosis, range: 2–9 months post-diagnosis). During the interview, information about their demographic and lifestyle characteristics were collected using a breast cancer survivorship questionnaire specific to the study. Participants consented to regular, prospective follow-up for up to 10 years, however, only data from the follow-up surveys at 2 years (median: 25 months, range: 20–36 months) and at 7 years (median: 84 months, range: 60–110 months) post-breast cancer diagnosis were relevant for the current analysis. Tumour characteristics (e.g., stage at diagnosis and grade) were extracted from pathology reports, while information about baseline comorbidities, breast cancer treatment, and type of surgery were extracted from medical records by chart review.

The current analysis excluded CBCS3 participants who did not have their first course of surgery within 18 months of breast cancer diagnosis (*n =* 49), but included data from three women whose baseline survey date preceded their first surgery. Additional exclusions included women who already had a self-reported diagnosis of lymphoedema by a health care provider before breast cancer surgery (*n =* 21), women who were diagnosed with stage IV breast cancer due to different treatment strategies compared with women diagnosed with stage I–III breast cancer (*n =* 109) [18], and women who had recurrent disease throughout the 7-year follow up period (*n =* 426).

### 2.1. Outcome Ascertainment

#### 2.1.1. Upper-Body Symptoms

Information about the presence and severity of upper-body symptoms on the treated side was collected using responses to specific symptom items within the validated Functional Assessment of Cancer Therapy- Breast cancer (FACT-B+4) (pain, poor range of movement, numbness, stiffness) [19] the Disabilities of the Arm, Shoulder and Hand (DASH) questionnaire (pain, tingling, weakness, stiffness) [20,21], and an additional three symptoms identified as relevant in formative work (heaviness, achiness, tightness) [7]. General pain and tingling from the DASH were the only symptoms assessed at 7-years post-diagnosis. Items from the FACTB+4, heaviness, achiness, and tightness, and items from the DASH asked participants to rate the severity of symptoms in the last week using a 5-point Likert scale (FACTB+4 items, heaviness, achiness and tightness: 0 = not at all, 1 = a little bit, 2 = somewhat, 3 = quite a bit, 4 = very much; DASH items: 0 = not present, 1 = mild, 2 = moderate, 3 = severe, 4 = extreme). Responses of ≥1 were used to indicate the presence of a symptom of at least mild severity, while responses of ≥2 indicated a symptom presence of at least moderate severity. 

#### 2.1.2. Upper-Body Function

The QuickDASH is a participant-reported, validated method of assessing upper-body function by capturing information about the level of difficulty experienced when performing specific tasks, the extent to which upper-body function interferes with normal activities and the severity of specific upper-body symptoms [20,21]. The QuickDASH includes 11 items with responses collected via a 5-point Likert scale. The total score ranges from 0 to 100, with lower scores reflecting better upper-body function. 

#### 2.1.3. Breast Cancer-Related Lymphoedema

BCRL was assessed via self-report of a clinical diagnosis of BCRL (yes/no; date of diagnosis) including the location of BCRL (left/right arm, trunk, or breast) and information on the health professional who provided the diagnosis (i.e., medical doctor, nurse, physical therapist, other). 

#### 2.1.4. Quality of Life

The FACTB+4 is a participant-reported, validated questionnaire that includes 37 items (responses collected using 5-point Likert scale) that enables the measurement of physical, social, emotional, and functional well-being as well as overall quality of life, a breast cancer subscale (B+4), and functional status (the Breast Cancer Trial Outcome Index: sum of physical well-being, functional well-being, and the breast cancer subscale) [19]. Higher scores indicate higher subscales and overall quality of life. 

#### 2.1.5. Physical Activity

Participant-reported total minutes of moderate-intensity or higher weekly physical activity pre-diagnosis (approximately 3 months before breast cancer diagnosis) and post-diagnosis (7 days before interview) were captured using items from the 2001 Behavioural Risk Factor Surveillance System survey [22]. Participants were then categorised as being sedentary, insufficiently active, or sufficiently active according to their total weekly physical activity levels (0 min, 1–150 min, >150 min, respectively) [23]. 

### 2.2. Statistical Analyses

The proportion of women reporting each symptom (12 in total including three items for pain, two items for stiffness, and one item for poor range of movement, numbness, heaviness, achiness, tightness, tingling, and weakness) of at least mild and at least moderate severity were calculated at each time point (baseline, and 2- and 7-years post-diagnosis). These data were then used to calculate (i) the proportion of women reporting the presence of 0–9 symptoms of at least mild severity, and of at least moderate severity (note that the ‘pain (general)’ and ‘stiffness (treated side)’ items were used to reflect pain and stiffness, respectively); and (ii) a dichotomous upper-body symptom variable that was created to report those with no symptoms versus those with one or more symptoms, at each time point. The relevant statistical tests (including Mann–Whitney tests, unpaired t-tests, and chi-squared analyses) were undertaken to explore the relationship between the presence of symptoms and upper-body function, BCRL, physical activity, and quality of life. We also explored the average number of symptoms reported by those with better versus poorer upper-body function (DASH score of <15 versus 15+) and the presence of BCRL (no versus yes) using unpaired t-tests. A score of 15 on the DASH was a priori considered meaningful as it indicates that a participant has at least mild difficulty in undertaking at least 50% of activities (items) assessed on the questionnaire. Quality of life (FACTB+4) data were normally distributed and therefore described using means and standard deviations, with a difference in 8, 5, 3, and 5 units a priori defined as clinically relevant for FACTB+4, FACTG, breast cancer subscale (B+4), and trial outcome index (physical, functional, and breast cancer subscale), respectively [24]. Medians (minimum, maximum and interquartile range) were used to describe nonparametric continuous outcomes including QuickDASH and physical activity data. A minimal clinically important difference of 15 units was determined a priori for the QuickDASH [25]. A difference of 20 min of moderate intensity physical activity per week was deemed as being clinically relevant for weekly physical activity data [21].

Unadjusted and adjusted logistic regression analyses were used to assess the relationship between upper-body symptoms at baseline and BCRL at 2- and 7-year follow-up. Covariates were identified based on a priori knowledge and associations in this study and included the age and stage of disease at diagnosis, number of lymph nodes removed, treatment type, self-identified race, body mass index, upper-body function, and pre- and post-diagnosis (as assessed at baseline) physical activity levels. All statistical tests were 2-sided and considered statistically significant at *p* < 0.05. Statistical analyses were performed using Statistical Analysis System (SAS) software (version 9.4; SAS Institute, Inc., Cary, NC, USA).

## 3. Results

Characteristics of the CBCS3 sample contributing to this work are presented in Table 1, and characteristics of those with complete BCRL data at baseline (*n =* 2442), and 2- (*n =* 2170) and 7- (*n =* 1698) years post-diagnosis versus those with missing BCRL data at 2- (*n =* 272) or 7- (*n =* 744) years post-diagnosis are presented in Table S1. There were higher proportions of white women, younger women (<50 years), women with stage II and III disease, and women who were classified as sedentary or insufficiently active at baseline in cases with missing BCRL data versus those whose BCRL status was known. 

Between 28–55% and 31–53% of women reported any given symptom at baseline and at 2-years post-breast cancer diagnosis of at least mild severity, respectively, while any given symptom of at least moderate severity was reported by 15–28% and 17–26% at baseline and 2-years post-diagnosis, respectively (Table 2). The median number of symptoms reported of at least mild severity at baseline was 3, and the median number of symptoms of at least moderate severity was 1. By 2 years post-diagnosis, the median number of symptoms of at least mild severity did not change (median: 3), while the median number of symptoms of at least moderate severity was 0. Approximately two in three women reported more than one symptom of mild severity at the baseline and at 2-years post-diagnosis, while one in three women reported more than one symptom of moderate severity. Compared with the prevalence at baseline and 2-years post-diagnosis, similar proportions of women reported general pain (mild severity or higher: N, 890; 53.1%; moderate severity or higher: N, 460, 27.4%) and tingling (mild severity or higher: N, 764; 45.9%; moderate severity or higher: N, 413, 24.8%) at 7-years post-diagnosis.

The presence of one or more symptoms of at least mild severity was associated (*p* < 0.01) with poorer upper-body function, higher prevalence of BCRL, lower levels of post-diagnosis weekly physical activity, higher proportions of sedentary women, lower proportions of sufficiently active women, and poorer overall quality of life and subscales. These relationships were consistently observed at the baseline, and at 2- and 7-years post-diagnosis (Table 3), and when only symptoms of moderate or higher severity were considered (Table S2). Furthermore, a higher number of upper-body symptoms were reported in those with poorer upper-body function and the presence of lymphoedema when compared with those who reported better upper-body function (*p* < 0.01) and no lymphoedema (*p* < 0.01), respectively (Table 4).

The odds ratios (ORs) of BCRL at 2- and 7-years post-diagnosis for those who report any given symptom (of mild severity or higher) at the baseline were generally higher than 1, although they ranged between 0.98 (stiffness) and 1.94 (tightness), with >50% of the relationships supported statistically (*p* < 0.05, Table S3). Only the presence of heaviness, achiness, or tightness was consistently associated with increased odds of BCRL at 2- and 7-years post-diagnosis, irrespective of whether the severity was at least of mild, or at least of moderate severity (all OR ≥1.5, *p* < 0.05). When adjusted for demographic and clinical BCRL risk factors, the ORs of developing BCRL by 2- and 7-years post-diagnosis increased with the higher the number of symptoms present at the baseline of at least moderate severity (all OR >1.27, Table 5). When symptoms of mild severity were also considered (Table S4), odds of BCRL at 2-years post-diagnosis were increased when 3 or more symptoms were present (OR: 1.92, *p* < 0.05), while 7 or more symptoms of at least mild severity at baseline were associated with increased odds of BCRL at 7-years post-diagnosis (OR: 2.15, *p* < 0.01, Table S4). 

Findings of our final model that included all potential confounders (demographic and clinical characteristics) represent the most conservative findings for the relationship between symptoms and breast cancer-related lymphoedema compared to the unadjusted model, the demographic model (including participant race, age, body mass index at baseline, pre- and post-diagnosis physical activity levels and baseline upper body function), and the clinical model (including participant cancer stage at diagnosis, number of extracted lymph nodes, and treatment type). All findings remained consistent in the sensitivity analyses that involved the removal of data from women who reported BCRL at the baseline assessment (results not shown).

## 4. Discussion

In the Carolina Breast Cancer Study, Phase 3, we found that the presence of upper-body symptoms including pain, poor range of movement, numbness, stiffness, heaviness, achiness, tightening, tingling or weakness is common following breast cancer. At least 28% of women, but up to 55%, reported the presence of at least one symptom within the first 9 months post-diagnosis. The majority of women (approximately two in three) reported two or more symptoms of at least mild severity. Symptoms remained common even when mild symptoms were not considered (that is, when only considering symptoms reported of at least moderate severity). Under these conditions, about one in two women reported one or more symptoms at the baseline and 2-years post-diagnosis. The findings also suggest that upper-body symptoms remained common well into breast cancer survivorship as symptom prevalence did not change between the baseline, and 2- and 7-years post-diagnosis for pain and tingling. The presence of upper-body symptoms was associated with poorer upper-body function, higher prevalence of BCRL, lower levels of weekly physical activity, and poorer quality of life. Furthermore, one or more symptoms of at least moderate severity at the baseline increased the odds of developing BCRL by 2- and 7-years post-diagnosis, and the higher the number of moderate to severe symptoms was associated with higher odds of developing BCRL. 

The symptom prevalence findings in this study were similar to those reported by a previous population-based, longitudinal study of 287 women diagnosed with breast cancer who were followed up until 18 months post-surgery, published more than a decade ago [7]. Another more recent prospective, longitudinal study in 486 women undergoing axillary lymph node dissection and neoadjuvant treatment reported a cumulative incidence of upper-body symptoms of 37.8% (95% confidence interval: 33.1%, 43.2%) at 3-years post-surgery [26]. Furthermore, findings from the secondary analysis of randomised, trial data from 508 women followed up until 24-months post-surgery also showed similar symptom prevalence patterns and demonstrated that symptom severity increased from the baseline to 6-months post-surgery and then remained steady up until 2-years post-surgery [27]. Based on these observations, advances in treatment methods over the past decades including less invasive surgery and more targeted radiation do not seem to be translating into reduced frequency or severity of upper-body symptoms in the short-or longer-term. 

These findings support that the presence of any given upper-body symptom (even when mild in severity) matters to the quality of breast cancer survivorship as symptoms were associated with poorer upper-body function, lower levels of physical activity, poorer quality of life, and higher rates of BCRL [10]. Furthermore, our findings suggest that the presence of symptoms at the baseline indicated increased odds of subsequently developing BCRL by 2-years post-diagnosis, with the presence of (i) heaviness, achiness, or tightness; (ii) one or more symptoms of moderate severity; or (iii) three or more symptoms of at least mild severity, all warranting clinical attention. Work by others has also demonstrated the relationship between upper-body symptoms and BCRL. For example, a population-based cross-sectional study of 1067 women who underwent axillary node dissection showed more prevalent, clinically relevant upper-body symptoms in women with BCRL compared to those who did not (measured up until 10-years after diagnosis), with increased lymphoedema severity being associated with more severe symptom presentations [10]. However, to our knowledge, our findings are the first to demonstrate the predictive relationship between upper-body symptoms and BCRL, and provide scientific evidence to support clinical observations and lymphoedema staging definitions that indicate that upper-body symptoms typically precede measurable swelling [5]. 

While upper-body symptoms were found to predict BCRL, it is also important to highlight that symptoms were common in women who did not have BCRL (that is, women who did not have BCRL reported on average the presence of three and two symptoms of at least mild severity, at the baseline and at 2-years post-diagnosis, respectively). Therefore, caution in using the presence of multiple mild symptoms to inform diagnosis of BCRL is warranted. Nonetheless, our findings also showed that it was uncommon for women without BCRL to report more than three symptoms of moderate to extreme severity (median number of symptoms at the baseline and 2-years post-diagnosis was 1 (interquartile range (IQR): 0, 4) and 0 (IQR: 0, 3), respectively). As such, these findings support that the reporting of four or more upper-body symptoms of moderate severity or higher, is likely an appropriate indicator of the need for further BCRL investigation, and the presence of one or more symptoms of any severity may justify the need for prospective surveillance and management. Current best practice in BCRL management involves early diagnosis through prospective surveillance, and subsequent treatment with compression, which has been shown to prevent BCRL progression [28,29,30]. However, prospective surveillance, particularly when it involves the use of objective BCRL assessments such as the use of circumferences, perometry, or bioimpedance spectroscopy is burdensome to an already stretched health care system and adds to disease management-related costs for patients. Therefore, it seems plausible that the presence and severity of upper-body symptoms could be used to identify those women who could benefit most from active BCRL lymphoedema surveillance, and avoid unnecessary follow up for those who do not report upper-body symptoms. 

The symptoms could also be used to identify those women who could benefit most from risk prevention strategies. In a recently published meta-analysis, exercise therapy involving aerobic and/or resistance exercise of moderate or higher intensity was shown to reduce BCRL incidence (relative risk: 0.49, *p* < 0.05, *n =* 6 studies, *n =* 552 participants) [14]. Our findings showed that those who reported symptoms also reported lower upper-body function and levels of physical activity when compared with those who did not report symptoms. These findings highlight the capacity for improvements in physical activity levels through exercise therapy, particularly for those reporting symptoms, and thereby possibly decreasing their risk of developing BCRL. Recently, published findings from a randomised, controlled trial also support the use of 6 months of compression in the prevention of BCRL [13]. However, women wearing compression in the treatment of BCRL report that compression garments are hot, restrict movement, are a visible reminder of having breast cancer, and increase financial burden (garments are expensive). A published review further identified that the most common non-severe medical compression therapy-associated adverse events included skin irritation, discomfort, and pain. Very rare but severe adverse events including soft tissue and nerve injury were also identified [31]. Consequently, the potential benefits alongside the potential risks of using compression to prevent BCRL need to be considered, particularly for women at low-risk of developing BCRL. Future BCRL prevention research could consider using symptom frequency and severity as part of the eligibility criteria.

The strengths of this work include a large, population-based, representative sample of women with breast cancer, and a prospective, comprehensive assessment of the upper-body symptoms. The use of data collected via individual items within validated questionnaires in the collection of upper-symptom data could be viewed as a limitation of this work. However, the participants completed the questionnaires in their validated format and in using items within existing questionnaires, we were able to reduce unnecessary participant burden while concurrently collecting comprehensive symptom data. Another potential limitation of this work was the self-reported assessment of BCRL, although findings from formative work indicated that similar findings were observed when BCRL was assessed using objective assessment (specifically, bioimpedance spectroscopy) and self-report [32], and we reduced the potential for over-reporting by asking the participants to self-report BCRL that had been diagnosed by a health professional. The large sample size provided adequate power for adjusted regression analyses and facilitated relatively tight confidence intervals, providing confidence in the estimated effect sizes. Furthermore, primary analyses used all available data at all time points, and results from the sensitivity analyses that excluded women with evidence of BCRL at the baseline did not influence the findings. Nonetheless, women who participated in this cohort study received their initial treatment for breast cancer over 10 years prior. Some changes have occurred in treatment patterns since that time, and although our final model was adjusted for potentially confounding clinical and demographic variables, there is the possibility that other life events may have interfered with this relationship that were not captured within the data. Excluding participants for whom we had no BCRL data at 2- and 7-years post-diagnosis (11% and 30%, respectively) may also have induced selection bias, with higher proportions of White and younger women, women with stage II/III disease at diagnosis, and active women; although this was likely in the conservative direction. Nonetheless, the study did include a large proportion of diverse women. 

## 5. Conclusions

In summary, our findings show that symptoms are common following breast cancer, persist long after the active treatment period, and are associated with poorer survivorship outcomes including reduced upper-body function, physical activity, and quality of life as well as increased odds of developing BCRL. Members of the multidisciplinary breast cancer care team need to be aware that the presence of even mild symptoms are relevant to other health outcomes and that the higher the number of upper-body symptoms, particularly symptoms of moderate severity or higher, increases the odds of developing BCRL. Future research that explores the role of symptom presence in risk stratification for BCRL prevention strategies is warranted.

## Figures and Tables

**Table 1:** Characteristics of the participants of the Carolina Breast Cancer Study, Phase III.

Characteristic	N (%)
Race	
Black	1289 (52.8%)
White	1153 (47.2%)
Age	
<50	1189 (48.7%)
50+	1253 (51.3%)
Stage at Diagnosis	
I	1141 (46.8%)
II	1006 (41.2%)
III	293 (12.0%)
Treatment Type	
Surgery Only	326 (13.4%)
Surgery + Radiation	617 (25.3%)
Surgery + Chemotherapy	348 (14.3%)
Surgery, Radiation, Chemotherapy	1151 (47.1%)
Body mass index	
<25	676 (27.8%)
25–30	1147 (47.2%)
30+	609 (25.0%)
Physical activity (3 months before diagnosis)	
Sedentary	386 (15.8%)
Insufficiently active	546 (22.4%)
Sufficiently active	1506 (61.8%)
Physical activity (median 5 months post-diagnosis)	
Sedentary	1062 (43.5%)
Insufficiently active	561 (23.0%)
Sufficiently active	816 (33.5%)
Number of lymph nodes examined median (min, max)	4 (0, 57)

**Table 2:** Upper-body symptoms reported by participants of the Carolina Breast Cancer Study, Phase III at baseline^a^ and 2-years post-breast cancer diagnosis (PD).

Upper-Body Symptoms	Indicated as at Least Mild in Severity		Indicated as at Least Moderate in Severity	
	Baseline ^a^	2-Years PD	Baseline ^a^	2-Years PD
	N (%)	N (%)	N (%)	N (%)
Pain with movement	1151 (47.2%)	959 (44.7%)	528 (21.7%)	429 (20.0%)
Pain (general)	1297 (53.3%)	1101 (51.5%)	597 (24.5%)	523 (24.5%)
Pain with specific activity	1327 (54.7%)	1135 (53.4%)	652 (26.9%)	560 (26.3%)
Poor range of arm movement	1028 (42.2%)	932 (43.6%)	487 (20.0%)	462 (21.6%)
Numbness	1045 (43.0%)	929 (43.3%)	668 (27.5%)	539 (25.1%)
Stiffness (treated side)	920 (37.8%)	828 (38.7%)	469 (19.3%)	415 (19.4%)
Stiffness (arm, shoulder, hand)	1122 (46.0%)	1055 (49.4%)	523 (21.5%)	490 (22.9%)
Heaviness	684 (28.1%)	658 (30.7%)	358 (14.7%)	368 (17.2%)
Achiness	990 (40.6%)	900 (42.0%)	520 (21.3%)	476 (22.2%)
Tightness	1052 (43.2%)	926 (43.2%)	563 (23.1%)	479 (22.4%)
Tingling	1155 (47.4%)	1008 (47.3%)	613 (25.2%)	513 (24.1%)
Weakness	1238 (50.8%)	1116 (52.3%)	578 (23.7%)	552 (25.9%)
Number of symptoms ^b^:				
Median (min, max)	3 (0, 9)	3 (0, 9)	1 (0, 9)	0 (0, 9)
0	567 (23.3%)	482 (22.8%)	1204 (49.6%)	1107 (52.3%)
1	277 (11.4%)	264 (12.5%)	332 (13.7%)	236 (11.2%)
2	215 (8.9%)	217 (10.3%)	201 (8.3%)	191 (9.0%)
3	182 (7.5%)	153 (7.2%)	128 (5.3%)	100 (4.7%)
4	188 (7.7%)	118 (5.6%)	113 (4.7%)	86 (4.1%)
5	164 (6.8%)	141 (6.7%)	96 (4.0%)	68 (3.2%)
6	149 (6.1%)	125 (5.9%)	79 (3.3%)	69 (3.3%)
7	166 (6.8%)	131 (6.2%)	90 (3.7%)	64 (3.0%)
8	197 (8.1%)	157 (7.4%)	82 (3.4%)	54 (2.6%)
9	324 (13.3%)	328 (15.5%)	104 (4.3%)	141 (6.7%)

PD, post-diagnosis; ^a^ Baseline: Median time of assessment was 5 months post-diagnosis; ^b^ Pain (general) and stiffness (treated side) were used as the contributing items for pain and stiffness, respectively; 13 and 54 with incomplete data to allow for categorising within this variable at the baseline and 2-years PD.

**Table 3:** Relationships between the upper-body symptoms of at least mild severity and upper-body function, breast cancer-related lymphoedema, physical activity, and quality of life up to 7-years post-diagnosis of breast cancer.

Timing of Assessment	Baseline ^a^			2-Years Post-Diagnosis			7-Years Post-Diagnosis ^b^		
Upper-body symptoms (at least one symptom of mild severity or higher)									
	**No**	**Yes**	***p* Value**	**No**	**Yes**	***p* Value**	**No**	**Yes**	***p* Value**
Upper-body function									
QuickDASH ^c^: median	2.3	22.7	<0.001	2.3	18.2	<0.001	2.3	29.5	<0.001
(min, max)	(0.0, 43.2)	(0.0, 97.7)		(0.0, 63.6)	(0.0, 97.7)		(0.0, 55.0)	(2.3, 100.0)	
IQR ^d^	(0.0, 9.1)	(11.4, 38.6)		(0.0, 4.5)	(6.8, 38.6)		(0, 6.8)	(15.9, 50.0)	
Breast cancer-related lymphoedema									
Prevalence N (%)	6 (1.1)	157 (8.4)	<0.001	22 (4.6)	414 (25.3)	<0.001	98 (12.5)	314 (35.3)	<0.001
Total physical activity (of moderate intensity or higher) as assessed at baseline ^a^, minutes/week									
median	90	30	<0.001	113	40	<0.001	90	10	<0.001
(min, max)	(0, 4200)	(0, 5040)		(0, 4200)	(0, 5040)		(0, 5040)	(0, 3690)	
IQR ^d^	(0, 300)	(0, 210)		(0, 360)	(0, 210)		(0, 280)	(0, 180)	
Sedentary, N (%)	203 (35.8)	855 (46.0)	<0.001	166 (34.4)	727 (44.6)	<0.001	255 (32.4)	442 (49.8)	<0.001
Insufficiently active N, (%)	137 (24.2)	421 (22.7)	<0.001	116 (24.1)	387 (23.7)	<0.001	211 (26.8)	193 (21.7)	<0.001
Sufficiently Active N, (%)	227 (40.0)	583 (31.4)	<0.001	200 (41.5)	518 (31.7)	<0.001	321 (40.8)	254 (28.5)	<0.001
Quality of life and subscales									
Lymphoedema (+4 subscale)	20.0 (0.3)	15.0 (4.5)	<0.001	19.9 (0.4)	15.0 (4.8)	<0.001			
Functional status (FACT TOI) ^e^	76.4 (13.3)	62.0 (17.2)	<0.001	83.0 (9.3)	66.9 (17.7)	<0.001	81.7 (10.1)	64.0 (17.5)	<0.001
Overall QoL (FACTG) ^e^	121.7 (16.9)	103.5 (22.6)	<0.001	129.0 (13.5)	107.6 (24.2)	<0.001	127.0 (14.8)	103.6 (24.3)	<0.001
Breast cancer QoL (FACTB+4) ^e^	141.7 (16.9)	118.5 (25.0)	<0.001	149.0 (13.5)	122.7 (27.2)	<0.001			

^a^ Baseline assessed up to 9 months post-diagnosis (median time of assessment: 5 months post-diagnosis); ^b^ Only data collected from assessing general pain contributed to the results related to 7-year post-diagnosis relationships; ^c^ QuickDASH, Disability of the Arm, Shoulder and Hand questionnaire, total score 0–100, lower score equals better function; ^d^ IQR, interquartile range; ^e^ FACT, Functional Assessment of Cancer Therapy questionnaire—TOI: trial outcome index = sum of physical, functional and breast cancer subscale; G, general = sum of physical, social, emotional and functional subscale; B+4 = sum of G and the breast cancer specific subscale—higher scores equal higher quality of life.

**Table 4:** Presence of upper-body symptoms (of mild severity or higher) for those with better versus lower upper-body function and those with and without breast cancer-related lymphoedema among participants in the Carolina Breast Cancer Study, Phase III.

Timing of Assessment	Baseline ^a^			2-Years Post-Diagnosis		
Upper-Body Function ^b^						
	**Better**	**Poorer**	***p* Value**	**Better**	**Poorer**	***p* Value**
Symptoms (at least mild in severity)						
Median (min, max) IQR ^c^	1 (0, 9) (0, 3)	6 (0, 9) (3, 8)	<0.001	1 (0, 9) (0, 3)	7 (0, 9) (0, 3)	<0.001
Symptoms (at least moderate in severity)						
median (min, max) IQR ^c^	0 (0, 7) (0, 0)	3 (0, 9) (1, 6)	<0.001	0 (0, 7) (0, 0)	3 (0, 9) (1, 7)	<0.001
Presence of breast cancer-related lymphoedema						
	**no**	**yes**	***p* Value**	**no**	**yes**	***p* Value**
Symptoms (at least mild in severity)						
Median (min, max) IQR ^c^	3 (0, 9) (1, 7)	8 (0, 9) (4, 9)	<0.001	2 (0, 9) (0, 6)	8 (0, 9) (4, 9)	<0.001
Symptoms (at least moderate in severity)						
Median (min, max) IQR ^c^	0 (0, 9) (0, 3)	3 (0, 9) (1, 7)	<0.001	0 (0, 9) (0, 2)	3 (0, 9) (0, 8)	<0.001

^a^ Baseline assessed up to 9 months post-diagnosis (median time of assessment: 5 months post-diagnosis); ^b^ <15 and 15+ on the Quick Disability of the Arm, Shoulder, and Hand questionnaire represented those with better versus poorer upper-body function. ^c^ IQR, interquartile range.

**Table 5:** Prevalence of breast cancer-related lymphoedema and odds ratio of lymphoedema at 2- and 7-years post-diagnosis breast cancer for those with 0, 1–2, 3–4, 5–6, 7–9 upper-body symptoms (of at least moderate severity) at the baseline among women in the Carolina Breast Cancer Study, Phase III.

Breast Cancer-Related Lymphoedema Prevalence at 2-Years Post-Diagnosis									
Number of Baseline ^a^ Symptoms	Lymphoedema Prevalence N (%)	Unadjusted Model (*n =* 2165) OR (95% CI)	*p* Value	Demographic Model ^b^ (*n =* 2150) OR (95% CI)	*p* value	Clinical Model ^c^ (*n =* 2163) OR (95% CI)	*p* Value	Full Model ^d^ (*n =* 2148) OR (95% CI)	*p* Value
0	135/1093 (12.4%)	1 (ref)		1 (ref)		1 (ref)		1 (ref)	
1–2	110/480 (22.9%)	2.11 (1.60–2.79)	<0.001	1.79 (1.33–2.42)	0.001	1.74 (1.30–2.34)	<0.001	1.49 (1.09–2.04)	0.013
3–4	63/211 (29.9%)	3.02 (2.14–4.27)	<0.001	2.26 (1.53–3.33)	<0.001	2.36 (1.63–3.40)	<0.001	1.75 (1.16–2.63)	0.008
5–6	39/149 (26.2%)	2.52 (1.67–3.78)	<0.001	1.76 (1.10–2.81)	0.018	1.88 (1.22–2.89)	0.004	1.31 (0.80–2.14)	0.284
7–9	104/226 (46.0%)	6.05 (4.40–8.31)	<0.001	3.37 (2.14–5.31)	<0.001	4.57 (3.26–6.42)	<0.001	2.58 (1.60–4.17)	<0.001
Breast cancer-related lymphoedema at 7-years post-diagnosis									
Number of baseline ^a^ symptoms	Lymphoedema Prevalence N (%)	Unadjusted Model (*n =* 1694) OR (95% CI)		Demographic Model ^b^ (*n =* 1684) OR (95% CI)		Clinical Model ^c^ (*n =* 1693) OR (95% CI)		Full model ^d^ (*n =* 1683) OR (95% CI)	
0	124/877 (14.1 %)	1 (ref)		1 (ref)		1 (ref)		1 (ref)	
1–2	100/372 (26.9%)	2.23 (1.66–3.01)	<0.001	1.83 (1.32–2.53)	<0.001	1.78 (1.30–2.44)	<0.001	1.45 (1.03–2.05)	0.032
3–4	65/165 (39.4%)	3.95 (2.74–5.69)	<0.001	2.86 (1.88–4.35)	<0.001	2.97 (2.01–4.39)	<0.001	2.07 (1.33–3.23)	0.001
5–6	32/105 (30.5%)	2.66 (1.69–4.20)	<0.001	1.92 (1.15–3.20)	0.013	1.80 (1.10–2.95)	0.019	1.27 (0.74–2.18)	0.388
7–9	94/171 (55.0%)	7.41 (5.19–10.58)	<0.001	3.94 (2.38–6.51)	<0.001	5.42 (3.69–7.95)	<0.001	2.75 (1.61–4.70)	<0.001

^a^ Baseline assessment occurred between 2 to 9 months post-diagnosis (median time of assessment: 5 months post-diagnosis); ^b^ Demographic model was adjusted for the participant’s race, age, BMI at baseline, and pre- and post-(baseline) diagnostic physical activity levels and baseline upper body function (as measured by QuickDASH); ^c^ Clinical model was adjusted for participant cancer stage at diagnosis, number of extracted lymph nodes, and treatment type (Surgery, surgery + radiation, surgery+ chemotherapy, or surgery + radiation + chemotherapy); ^d^ The full model was adjusted for covariates in both the demographic and clinical models. CI, confidence interval; OR, odds ratio.

## Data Availability

Data supporting the reported results can be made available upon request.

## Supplementary Materials

The following supporting information can be downloaded at: https://www.mdpi.com/article/10.3390/cancers14235998/s1, Table S1: Characteristics of those in the Carolina Breast Cancer Study, Phase 3 for whom we have breast cancer-related lymphoedema outcome data at the baseline and 2- and 7-years post-diagnosis as well as for those for whom breast cancer-related lymphoedema data are missing at the 2- or 7-year post-diagnosis follow-up; Table S2: Relationships between the upper-body symptoms (of at least moderate severity) and upper-body function, breast cancer-related lymphoedema, physical activity, and quality of life up to 7-years post-diagnosis of breast cancer; Table S3: Odds of having breast cancer-related lymphoedema at 2- and 7-years post-diagnosis for those with any given upper-body symptom (of at least mild severity, and at least moderate severity) at baseline ^a^ among women in the Carolina Breast Cancer Study, Phase 3; Table S4: Prevalence of breast cancer-related lymphoedema and the odds ratio of lymphoedema at 2- and 7-years post-diagnosis breast cancer for those with 0, 1–2, 3–4, 5–6, 7–9 upper-body symptoms (of at least mild severity) at the baseline ^a^ among women in the Carolina Breast Cancer Study, Phase 3.
