# Supplementary material for: Do Patient-Reported Upper-Body Symptoms Predict Breast Cancer-Related Lymphoedema: Results from a Population-Based, Longitudinal Breast Cancer Cohort Study
Source: Cancers (Basel). 2022 Dec 5;14(23):5998. doi: 10.3390/cancers14235998 (PMC9740941; doi:10.3390/cancers14235998)
Supplement: Supplementary file 1 [file cancers-14-05998-s001.zip › cancers-2044335-supplementary.pdf]

**Table S1.** Characteristics of those in the Carolina Breast Cancer Study Phase 3 for whom we have breast cancer-related lymphoedema outcome data at baseline and 2- and 7-years post-diagnosis, as well as for those for whom breast cancer-related lymphoedema data is missing at the 2- or 7-year post-diagnosis follow-up.

| | Baseline | 2-year post-diagnosis | | 7-year post-diagnosis | |
|---|---|---|---|---|---|
| Characteristic (N%) | BCRL known (n=2,442) | BCRL known (n=2170) | BCRL missing (n=272) | BCRL known (n=1,698) | BCRL missing (n=744) |
| Race | | | | | |
| Black | 1289 (52.8%) | 1174 (54.1%) | 115 (42.3%)** | 925 (54.5%) | 364 (48.9%)* |
| White | 1153 (47.2%) | 996 (45.9%) | 157 (57.7%) | 773 (45.5%) | 380 (51.1%) |
| Age | | | | | |
| <50 | 1189 (48.7%) | 1008 (46.5%) | 181 (66.5%)** | 788 (46.4%) | 401 (53.9%)* |
| 50+ | 1253 (51.3%) | 1162 (53.6%) | 91 (33.5%) | 910 (53.6%) | 343 (46.1%) |
| Stage at Diagnosis | | | | | |
| I | 1141 (46.8%) | 1044 (48.1%) | 97 (35.9%)** | 831 (48.9%) | 310 (41.8%)** |
| II | 1006 (41.2%) | 876 (40.4%) | 130 (48.2%) | 679 (40.0%) | 327 (44.1%) |
| III | 293 (12.0%) | 250 (11.5%) | 43 (15.9%) | 188 (11.1%) | 105 (14.2%) |
| Treatment Type | | | | | |
| Surgery Only | 326 (13.4%) | 281 (13.0%) | 45 (16.5%) | 207 (12.2%) | 119 (16.0%)* |
| Surgery, Radiation | 617 (25.3%) | 565 (26.0%) | 52 (19.1%) | 456 (26.9%) | 161 (21.6%) |
| Surgery, Chemotherapy | 348 (14.3%) | 305 (14.1%) | 43 (15.8%) | 240 (14.1%) | 108 (14.5%) |
| Surgery, Radiation, Chemotherapy | 1151 (47.1%) | 1019 (47.0%) | 132 (48.5%) | 795 (46.8%) | 356 (47.9%) |
| Body mass index | | | | | |
| <25 | 676 (27.8%) | 595 (27.6%) | 81 (29.8%) | 472 (27.9%) | 204 (27.5%) |
| 25-30 | 1147 (47.2%) | 1009 (46.7%) | 138 (50.7%) | 778 (46.0%) | 369 (49.8%) |
| 30+ | 609 (25.0%) | 556 (25.7%) | 53 (19.5%) | 441 (26.1%) | 168 (22.7%) |
| Physical activity (3 months before diagnosis) | | | | | |
| Sedentary | 386 (15.8%) | 322 (14.9%) | 64 (23.6%)** | 234 (13.8%) | 152 (20.5%)** |
| Insufficiently active | 546 (22.4%) | 488 (22.5%) | 58 (21.4%) | 374 (22.0%) | 172 (23.2%) |
| Sufficiently active | 1506 (61.8%) | 1357 (62.6%) | 149 (55.0%) | 1089 (64.2%) | 417 (56.3%) |
| Physical activity (median 5 months post-diagnosis) | | | | | |
| Sedentary | 1062 (43.5%) | 920 (42.4%) | 142 (52.4%)** | 706 (41.6%) | 356 (47.9%)* |
| Insufficiently active | 561 (23.0%) | 515 (23.8%) | 46 (17.0%) | 408 (24.1%) | 153 (20.6%) |
| Sufficiently active | 816 (33.5%) | 733 (33.8%) | 83 (30.6%) | 582 (34.3%) | 234 (31.5%) |
| Number of lymph nodes examined: median (min, max) | 4 (0, 57) | 4 (0, 98) | 5 (0,99) | 4 (0,97) | 4 (0,99) |

*p<0.05; **p<0.01.

**Table S2.** Relationships between upper-body symptoms (of at least moderate severity), and upper-body function, breast cancer-related lymphoedema, physical activity and quality of life up to 7-years post-diagnosis of breast cancer.

| Timing of assessment | Baseline [a] | | 2-years post-diagnosis | | 7-years post-diagnosis [b] | |
|---|---|---|---|---|---|---|
| Upper-body symptoms (at least one symptom of moderate severity or higher) | | | | | | |
| | **no** | **yes** | **no** | **yes** | **no (pain only)** | **yes (pain only)** |
| Upper-body function | | | | | | |
| QuickDASH[c]: median | 6.8 | 31.8*** | 4.5 | 29.5*** | 6.8 | 47.7*** |
| (min, max) | (0.0, 70.5) | (0.0, 97.7) | (0.0, 63.6) | (0.0, 97.7) | (0.0, 72.7) | (6.8, 100.0) |
| IQR [c] | (2.3, 15.0) | (18.2, 47.7) | (0, 11.4) | (15.9, 47.7) | (2.3, 15.9) | (34.1, 61.4) |
| Breast cancer-related lymphoedema | | | | | | |
| Prevalence N (%) | 39 (3.2)*** | 124 (10.1) | 118 (10.7)*** | 318 (31.5) | 218 (17.9)*** | 194 (42.2) |
| Pre-diagnosis minutes/week of total physical activity (of moderate severity or higher) | | | | | | |
| median | 240 | 210* | 240 | 210* | 240 | 210* |
| (min, max) | (0, 5400) | (0, 4620) | (0, 5400) | (0, 5070) | (0, 5400) | (0, 4260) |
| IQR [d] | (90, 600) | (60, 540) | (90, 560) | (70, 585) | (90, 600) | (60,540) |
| Sedentary, N (%) | 165 (13.6) | 218 (17.9)* | 136 (12.3) | 175 (17.4)** | 149 (12.3) | 81 (17.6)** |
| Insufficiently active N, (%) | 263 (21.8) | 280 (22.9)* | 243 (22.0) | 234 (23.2)** | 258 (21.2) | 113 (24.6)** |
| Sufficiently Active N, (%) | 776 (64.5) | 723 (59.2)* | 726 (65.7) | 599 (59.4)** | 809 (66.5) | 266 (57.8)** |
| Total physical activity (of moderate intensity or higher) as assessed at baseline[a], minutes/week | | | | | | |
| median | 85 | 0*** | 90 | 15*** | 90 | 0*** |
| (min, max) | (0, 5040) | (0, 4620) | (0, 5040) | (0, 4620) | (0, 5040) | (0, 3690) |
| IQR [d] | (0, 278) | (0,180) | (0, 280) | (0, 180) | (0, 240) | (0,120) |
| Sedentary, N (%) | 445 (37.0) | 613 (50.2)*** | 397 (359) | 496 (49.3)*** | 438 (36.0) | 259 (56.6)*** |
| Insufficiently active N, (%) | 301 (25.0) | 257 (21.0)*** | 290 (26.2) | 213 (212)*** | 313 (25.7) | 91 (19.9)*** |
| Sufficiently Active N, (%) | 458 (38.0) | 352 (28.8)*** | 420 (37.9) | 298 (30.0)*** | 466 (38.3) | 108 (23.6)*** |
| Quality of life and subscales | | | | | | |
| Lymphoedema (+4 subscale) | 19.1 (1.4) | 13.2 (4.6)*** | 19.1 (1.4) | 12.9 (4.9)*** | | |
| Functional status (FACT TOI) [e] | 73.7 (13.5) | 57.2 (17.0)*** | 79.4 (11.2) | 60.8 (18.0)*** | 78.4 (12.0) | 55.9 (17.4)*** |
| Overall QoL (FACTG) [e] | 118.3 (17.6) | 97.5 (22.5)*** | 123.9 (16.1) | 100.0 (24.9)*** | 122.7(17.2) | 93.2 (24.5)*** |
| Breast cancer QoL (FACTB+4) [e] | 137.3 (17.9) | 110.7(24.6)*** | 143.0 (16.5) | 112.9 (27.7)*** | | |

[a] Baseline assessment occurred between 2- to 9-months post-diagnosis (median time of assessment: 5 months post-diagnosis); [b] Only data collected from assessing general pain contributed to results related to 7-year post-diagnosis relationships; [c] QuickDASH, Disability of the Arm, Shoulder and Hand questionnaire, total score 0-100, lower score equals better function; [d] IQR, interquartile range; [e] FACT, Functional Assessment of Cancer Therapy questionnaire – TOI: trial outcome index = sum of physical, functional and breast cancer subscale; G, general = sum of physical, social, emotional and functional subscale; B+4 = sum of G and the breast cancer specific subscale – higher scores equal higher quality of of life; *p value< 0.05; **p value<0.01; ***p value<0.001.

**Table S3.** Odds of having breast cancer-related lymphoedema at 2- and 7-years post-diagnosis for those with any given upper-body symptom (of at least mild severity, and at least moderate severity) at baseline [a] among women in the Carolina Breast Cancer Study Phase 3.

| | Breast cancer-related lymphoedema at 2-years post-diagnosis (n=2172) [b] | | Breast cancer-related lymphoedema at 7-years post-diagnosis (n=1694) [b] | |
|---|---|---|---|---|
| | Symptom severity of mild or higher OR (95% CI) | Symptom severity of moderate or higher OR (95% CI) | Symptom severity of mild or higher OR (95% CI) | Symptoms severity of moderate or higher OR (95% CI) |
| **Symptom at baseline** [a] | | | | |
| Pain with movement | 1.47 (1.13-1.90) | 1.36 (1.00-1.84) | 1.18 (0.89-1.56) | 1.11 (0.79-1.56) |
| Pain (general) | 1.63 (1.22-2.20) | 1.06 (0.75-1.49) | 1.45 (1.06-1.99) | 1.03 (0.70-1.51) |
| Pain with specific activity | 1.29 (0.96-1.73) | 1.04 (0.74-1.47) | 1.08 (0.79-1.49) | 1.21 (0.83-1.76) |
| Poor range of arm movement | 1.58 (1.22-2.04) | 1.35 (1.00-1.84) | 1.13 (0.85-1.50) | 1.33 (0.94-1.87) |
| Numbness | 1.50 (1.17-1.94) | 1.45 (1.12-1.89) | 1.26 (0.96-1.65) | 1.22 (0.91-1.64) |
| Stiffness (side) | 1.63 (1.25-2.11) | 1.23 (0.91-1.67) | 1.36 (1.02-1.80) | 1.36 (0.98-1.90) |
| Heaviness | 1.70 (1.30-2.23) | 1.58 (1.15-2.18) | 1.71 (1.28-2.29) | 1.74 (1.22-2.47) |
| Achiness | 1.56 (1.20-2.02) | 1.50 (1.11-2.03) | 1.55 (1.16-2.05) | 1.70 (1.22-2.36) |
| Tightness | 1.86 (1.44-2.39) | 1.73 (1.31-2.28) | 1.94 (1.47-2.55) | 1.70 (1.25-2.31) |
| Tingling | 1.23 (0.95-1.60) | 0.98 (0.72-1.33) | 1.34 (1.01-1.77) | 1.26 (0.90-1.76) |
| Weakness | 1.37 (1.03-1.83) | 1.10 (0.78-1.55) | 1.53 (1.12-2.07) | 1.13 (0.77-1.65) |
| Stiffness (arm, shoulder, hand) | 1.18 (0.90-1.55) | 1.24 (0.89-1.74) | 1.10 (0.81-1.48) | 1.55 (1.06-2.27) |

[a] Baseline assessment occurred up to 9 months post-diagnosis (median time of assessment: 5 months post-diagnosis); [b] 4 women are missing baseline symptom data, and all models adjusted for participant race, age, body mass index at baseline, pre- and post-diagnostic physical activity levels, cancer stage at diagnosis, number of extracted lymph nodes, treatment type (surgery, surgery+radiation, surgery+chemotherapy, or surgery+radiation+chemotherapy) and upper-body function (as assessed by QuickDASH) for the symptoms model or number of symptoms (graded as mild severity or higher) for the upper-body function models. OR, odds ratio; CI, confidence interval.

**Table S4**. Prevalence of breast cancer-related lymphoedema and odds ratio of lymphoedema at 2- and 7-years post-diagnosis breast cancer for those with 0, 1-2, 3-4, 5-6, 7-9 upper-body symptoms (of at least mild severity) at baseline among women in the Carolina Breast Cancer Study Phase 3.

| Breast cancer-related lymphoedema at 2 years post-diagnosis | | | | | |
|---|---|---|---|---|---|
| Number of baseline [a] symtoms | Lymphoedema Prevalence N (%) | Unadjusted Model (n=2165) OR (95% CI) | Demographic Model [b] (n=2150) OR (95% CI) | Clinical Model [c] (n=2163) OR (95% CI) | Full model [d] (n=2148) OR (95% CI) |
| 0 | 43/125 (8.2%) | 1 (ref) | 1 (ref) | 1 (ref) | 1 (ref) |
| 1-2 | 59/441 (13.4%) | 1.73 (1.14-2.62)** | 1.51 (0.99-2.31) | 1.52 (0.99-2.34) | 1.32 (0.85-2.06) |
| 3-4 | 70/331 (21.2%) | 3.01 (2.00-4.52)*** | 2.50 (1.64-3.82)*** | 2.32 (1.52-3.55)** | 1.92 (1.23-2.98)** |
| 5-6 | 64/279 (22.9%) | 3.34 (2.20-5.07)*** | 2.61 (1.68-4.06)*** | 2.58 (1.66-3.99)*** | 2.01 (1.27-3.19)** |
| 7-9 | 215/583 (36.9%) | 6.55 (4.59-9.33)*** | 4.13 (2.70-6.30)*** | 4.55 (3.13-6.61)*** | 2.88 (1.84-4.49)*** |

| Breast cancer-related lymphoedema at 7 years post-diagnosis | | | | | |
|---|---|---|---|---|---|
| Number of baseline[a] symtoms | Lymphoedema Prevalence N (%) | Unadjusted Model (n=1694) OR (95% CI) | Demographic Model [b] (n=1684) OR (95% CI) | Clinical Model [c] (n=1693) OR (95% CI) | Full model [d] (n=1683) OR (95% CI) |
| 0 | 43/414 (10.4%) | 1 (ref) | 1 (ref) | 1 (ref) | 1 (ref) |
| 1-2 | 58/352 (16.5%) | 1.70 (1.11-2.60)* | 1.44 (0.93-2.23) | 1.43 (0.92-2.23) | 1.19 (0.75-1.87) |
| 3-4 | 66/266 (17.8%) | 2.85 (1.87-4.34)*** | 2.20 (1.41-3.42)** | 2.00 (1.28-3.11)** | 1.50 (0.94-2.39) |
| 5-6 | 58/219 (26.5%) | 3.11 (2.01-4.81)*** | 2.18 (1.37-3.46)** | 2.20 (1.39-3.48)** | 1.49 (0.91-2.44) |
| 7-9 | 190/439 (43.3%) | 6.58 (4.56-9.51)*** | 3.52 (2.25-5.51)*** | 4.19 (2.84-6.18)*** | 2.15 (1.34-3.46)** |

[a] Baseline assessment occurred between 2- and 9-months post-diagnosis (median time of assessment: 5 months post-diagnosis); [b] Demographic model is adjusted for participant race, age, body mass index at baseline, pre- and post-diagnostic physical activity levels and baseline upper body function (as

measured by QuickDASH); [c] Clinical model is adjusted for participant cancer stage at diagnosis, number of extracted lymph nodes, and treatment type (surgery, surgery+radiation, surgery+chemotherapy, or surgery+radiation+chemotherapy); [d] The full model is adjusted for covariates in both the demographic and clincal models. CI, confidence interval; OR, odds ratio. * p value<0.05; **p value<0.01; ***p value<0.001.
